# Supplementary material for: Information retrieval interfaces in virtual reality—A scoping review focused on current generation technology
Source: PLoS One. 2021 Feb 5;16(2):e0246398. doi: 10.1371/journal.pone.0246398 (PMC7864671; doi:10.1371/journal.pone.0246398)
Supplement: Attachment — Submitted filename: Response_to_Reviewers.pdf [file pone.0246398.s003.pdf]

Friday, January 8, 2021

Dear Dr. Ashra,
Dear reviewers,

Thank you for your message regarding my submission and the valuable feedback from the editor and both reviewers which I will gladly address.
The reviewers raised valid and important points and allowed me to improve my work. I am positive that I was able to respond to each of these points and implement changes in my revised submission. In the following, I will repeat each point in the same order as in your message (grey text) and explain how changes were implemented accordingly (black text).

I thank you for considering my work for resubmission and again for your time and feedback.

Sincerely,

Maurice Schleußinger, M.A.
Researcher, Department of Information Science
Heinrich Heine University Düsseldorf

Feedback from the Editor

Please include the following items when submitting your revised manuscript:

- A rebuttal letter that responds to each point raised by the academic editor and reviewer(s). You should upload this letter as a separate file labeled 'Response to Reviewers'.
- A marked-up copy of your manuscript that highlights changes made to the original version. You should upload this as a separate file labeled 'Revised Manuscript with Track Changes'.
- An unmarked version of your revised paper without tracked changes. You should upload this as a separate file labeled 'Manuscript'.

The three items were uploaded with the appropriate names in the Editorial Manager.

Journal Requirements:
When submitting your revision, we need you to address these additional requirements.

1. Please ensure that your manuscript meets PLOS ONE's style requirements, including those for file naming. The PLOS ONE style templates can be found at https://journals.plos.org/plosone/s/file?id=wjVg/PLOSOne_formatting_sample_main_body.pdf and https://journals.plos.org/plosone/s/file?id=ba62/PLOSOne_formatting_sample_title_authors_affiliations.pdf

I have adjusted the file names of the supporting files and the text is now formatted with double spacing. My manuscript was created with LaTeX using the provided template (https://journals.plos.org/plosone/s/latex). Please let me know if additional changes are required.

2. In your study selection flowchart (fig. 3), please specify the reasons for exclusion at each step of selection.

TODO Add final numbers after Reviewers feedback

3.Thank you for stating the following in the Funding Section of your manuscript:
[I acknowledge support by the Heinrich Heine University Duesseldorf.]
We note that you have provided funding information that is not currently declared in your Funding Statement. However, funding information should not appear in the Acknowledgments section or other areas of your manuscript. We will only publish funding information present in the Funding Statement section of the online submission form. Please remove any funding-related text from the manuscript and let us know how you would like to update your Funding Statement. Currently, your Funding Statement reads as follows:
[The author received no specific funding for this work.]

Please include your amended statements within your cover letter; we will change the online submission form on your behalf.

First, allow me to clarify the nature of the aforementioned statement. As funding for Open Access publications like PLOS One would be provided by my university library I am then required to add the statement "I acknowledge support by the Heinrich Heine University Düsseldorf". Thank you for clarifying the proper place to do so. I have removed the statement from my manuscript and updated my cover letter accordingly.

4. Your abstract cannot contain citations. Please only include citations in the body text of the manuscript, and ensure that they remain in ascending numerical order on first mention.

I have removed any citations from the abstract.

Feedback from the Reviewers

1. Is the manuscript technically sound, and do the data support the conclusions?

The manuscript must describe a technically sound piece of scientific research with data that supports the conclusions. Experiments must have been conducted rigorously, with appropriate controls, replication, and sample sizes. The conclusions must be drawn appropriately based on the data presented.
Reviewer #1: Yes
Reviewer #2: Yes

No changes required.

2. Has the statistical analysis been performed appropriately and rigorously?
Reviewer #1: Yes
Reviewer #2: Yes

No changes required.

3. Have the authors made all data underlying the findings in their manuscript fully available?

The PLOS Data policy requires authors to make all data underlying the findings described in their manuscript fully available without restriction, with rare exception (please refer to the Data Availability Statement in the manuscript PDF file). The data should be provided as part of the manuscript or its supporting information, or deposited to a public repository. For example, in addition to summary statistics, the data points behind means, medians and variance measures should be available. If there are restrictions on publicly sharing data—e.g. participant privacy or use of data from a third party—those must be specified.
Reviewer #1: No
Reviewer #2: Yes

TODO The full dataset was already submitted as supporting information. Additionally, I now also uploaded the initial dataset to the Zenodo repository. Please let me know how I should cite the repository entry.

4. Is the manuscript presented in an intelligible fashion and written in standard English?

PLOS ONE does not copyedit accepted manuscripts, so the language in submitted articles must be clear, correct, and unambiguous. Any typographical or grammatical errors should be corrected at revision, so please note any specific errors here.
Reviewer #1: Yes
Reviewer #2: Yes

No changes required.

5. Review Comments to the Author

Please use the space provided to explain your answers to the questions above. You may also include additional comments for the author, including concerns about dual publication, research ethics, or publication ethics. (Please upload your review as an attachment if it exceeds 20,000 characters)

Reviewer #1: The author provided a solid study on interactive information retrieval systam in Virtual Reality following the announcement and release of the current generation hardware system. It pre-designed four questions, covering the prototype/concepts, user interaction, retrieval features, and synthesis/display. After analysis a mount of related works, it present solid study results in corresponding to those questions. The data itself is solid and comprehensive. Moreover, it provided all the annotated data in the attachment. Consequently, I prefer to accept it for publication.

No changes required.

Reviewer #2: This article is an analysis of the state of the art related to Information Retrieval Interfaces in Virtual Reality. The manuscript is well organized and comprehensively described and with adequate references. In a high level, the paper tries to answer to five questions. The reviewer appreciates the effort and time spent for collecting and reviewing literature on the use of VR IR systems. However, the reviewer thinks the paper should be improved:

See following paragraphs.

- In the abstract mentioning just the Google Scholar, Scopus and Web of Science could be problematic because it is not clear why IEEE and ACM where not included.

I understand what the reviewer means and agree that this should be explained, but I believe it is helpful to only briefly mention the queried databases in the abstract without detailing the reasons for the exclusion of other databases. An explanation for the exclusion of both IEEE and ACM can already be found in the methods section (subsection *Information sources*): IEEE and ACM publications are being indexed in Google Scholar and are therefore fully included in this review. Additionally, early exploratory queries yielded no further relevant results.

- Related to the keywords, other terms could be incorporated, like “Head Mounted Display”

The reviewer raises a valid point. And while exploratory database searches with other keywords were conducted prior to the review, this wasn’t mentioned in the article. Therefore, I added details about both the thought process behind the final queries and the exploratory searches to the method section of the article (subsection *Search*). For example, database searches with variations for the query *"head mounted display" AND "information retrieval”* either yielded results that also included the more common terms *Augmented Reality*, *Virtual Reality* or *Mixed Reality* in either the title, abstract or as keywords while the other results were not relevant to the research questions. Additionally, the terms that are used in the queries are the most commonly accepted and used terms and are part of controlled vocabularies. Finally, by also analyzing through all works cited in relevant results and the additionally work from relevant authors I am confident that the keywords used for this review are sufficient and allowed a thorough scan of the body of relevant research.

- The introduction needs to focus on the topic and goals/contribution of present research. I think that explaining the concept and history of “Virtual Reality” is too much. This is not a survey of general VR technology.

I have removed the part from the introduction where more of a historical introduction is given. Additionally, I removed some of the more detailed definitions/explanations from the remaining introduction.

- In Methods: More of information about PRISMA should be included. Please specify what it represents and how it benefits this study. What is the reason for including student theses?

I have added a more detailed explanation to the Introduction (Rationale) to explain the importance of both systematic (scoping) reviews in general and the PRISMA guidelines specifically. I also highlighted how the PRISMA-ScR checklist builds on PRISMA and why and how it was applied to the given study.
I have added a short paragraph with reasons for including student theses to the method section. To summarize: Some student theses may accompany (or detail) peer-reviewed work. Finally, student theses of poor quality would be excluded or evaluated firstly by the author and secondly by the reader.

- In the Synthesis of results section review also the challenges and their implications in using VR IR systems compared to traditional (non-VR) IR systems.

I have added such a review to the Synthesis of results section in conjunction with RQ5. This includes an overview of the impressions in the topic that were given by the reviewed works as well as additional works. While I see potential for more detailed analysis, it is my understanding that there is a limit to the conclusions that can be reached based on the reviewed works and other literature at this point.

- The English language and style is good and clear to be understood.

No changes required.

Additional remarks:

- I have added citations to table 2 for easier comparison and stringency
- I have rephrased the last sentences of the abstract to be more precise
- The numbers on the PRISMA flow diagram and the dataset have been updated as some entries were counted multiple times (duplicates from different databases)
- Small corrections throughout the text
